# Phosphatases: Decoding the Role of Mycorrhizal Fungi in Plant Disease Resistance

**DOI:** 10.3390/ijms25179491

**Published:** 2024-08-31

**Authors:** Li Chen, Xiaoping Zhang, Qiang Li, Xuezhen Yang, Yu Huang, Bo Zhang, Lei Ye, Xiaolin Li

**Affiliations:** 1 Sichuan Institute of Edible Fungi, Sichuan Academy of Agricultural Sciences, Chengdu 610066, China; cl202301@126.com (L.C.); xiaopingzhang1997@outlook.com (X.Z.); sheboy111@163.com (X.Y.); yutou1227@yeah.net (Y.H.); bozhang5658@foxmail.com (B.Z.); yeleidewangyi@163.com (L.Y.); 2 College of Food and Biological Engineering, Chengdu University, Chengdu 610106, China; liqiang02@cdu.edu.cn

**Keywords:** mycorrhizal fungi, phosphatase, plant disease resistance, signal transduction, transcription factors

## Abstract

Mycorrhizal fungi, a category of fungi that form symbiotic relationships with plant roots, can participate in the induction of plant disease resistance by secreting phosphatase enzymes. While extensive research exists on the mechanisms by which mycorrhizal fungi induce resistance, the specific contributions of phosphatases to these processes require further elucidation. This article reviews the spectrum of mycorrhizal fungi-induced resistance mechanisms and synthesizes a current understanding of how phosphatases mediate these effects, such as the induction of defense structures in plants, the negative regulation of plant immune responses, and the limitation of pathogen invasion and spread. It explores the role of phosphatases in the resistance induced by mycorrhizal fungi and provides prospective future research directions in this field.

## 1. Introduction

Plants in the natural world are continually under the threat of pathogens, which can cause diseases that affect crop yield and quality. To counter these biotic stresses, plants have evolved a complex immune system.

**Figure 1:**
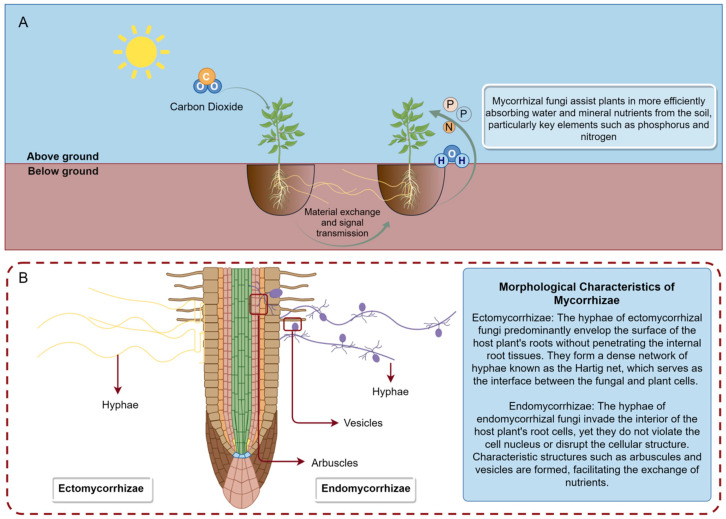
The functions of mycorrhizal fungi. (**A**) In the mycorrhizal symbiotic system, the organic carbon produced by plants through photosynthesis is primarily in the form of carbohydrates, such as glucose, fructose, and sucrose, which are transferred to mycorrhizal fungi via plant roots. In return, mycorrhizal fungi assist plants in more effectively absorbing water and mineral nutrients from the soil, particularly key elements like phosphorus and nitrogen. Additionally, mycorrhizal fungi form an extensive underground network through their hyphae, connecting different plant root systems to create a shared mycorrhizal network. Through this network, mycorrhizal fungi can engage in material exchange and signal transmission. (**B**) Comparative visualization of ectomycorrhizal and endomycorrhizal mycelia: ectomycorrhizal hyphae depicted on the left, with endomycorrhizal hyphae illustrated on the right.

The German scholar Frank discovered that the roots of some plants form symbiotic relationships with fungi. These fungi, while obtaining carbohydrates from the plants, help the plants absorb mineral elements and water from the soil. This symbiotic relationship is known as mycorrhiza [1]. Based on morphological characteristics, mycorrhizae can be mainly divided into ectomycorrhizae and endomycorrhizae. Among them, arbuscular mycorrhizae represent a special form of endomycorrhizae which are formed by *Glomeromycetes* fungi. These fungi play a vital role in enhancing the survival rate of plant seedlings [2], promoting plant growth [3], bioremediation [4], nutrient absorption [5,6], and increasing plant disease resistance [7,8] (Figure 1).

Phosphorus (P), as an essential nutrient element for plant growth, is mainly absorbed and utilized by plants in the form of inorganic phosphates (e.g., HPO_4_^2−^, H_2_PO_4_^−^) [9]. It also participates in various physiological and biochemical metabolic processes within the plant body, including energy transfer, signal transduction, redox reactions, and photosynthesis [10]. Improving the plant utilization of organic and insoluble inorganic phosphorus from the soil has been a longstanding agricultural challenge. Research indicates that, in addition to modulating the morphology and physiological characteristics of their root systems, plants have evolved a variety of mechanisms to cope with phosphorus deficiency, one of which is the formation of mycorrhizal symbioses to enhance phosphorus uptake from the soil [11]. Mycorrhizal fungi form a physical connection with plant roots through their extramatrical mycelium and extend in the soil to increase the plant’s absorption area for water and nutrients. Among them, the phosphatases secreted by mycorrhizal fungi can transform organic phosphorus in the soil into inorganic phosphorus that can be directly absorbed by plants, thereby improving the nutritional status of the plants. In addition, phosphatases may also enhance the plant’s defense against pathogens [12] by regulating the levels of plant hormones, activating plant defense-related genes [13], and enhancing systemic acquired resistance (SAR) [14,15,16,17].

This review aims to provide a comprehensive analysis of the biological functions of phosphatases in mycorrhizal fungi and their key roles in the plant disease defense mechanism, in order to deeply understand their molecular mechanisms and regulatory networks. By discussing the diversity and specificity of these key enzyme classes, this study aims to provide a theoretical basis and practical guidance for the development of new plant disease management strategies.

## 2. Mycorrhizal Fungi-Induced Host Plant Resistance to Disease

### 2.1. The Array of Plant Pathologies That Mycorrhizal Fungi Can Suppress

**Figure 2:**
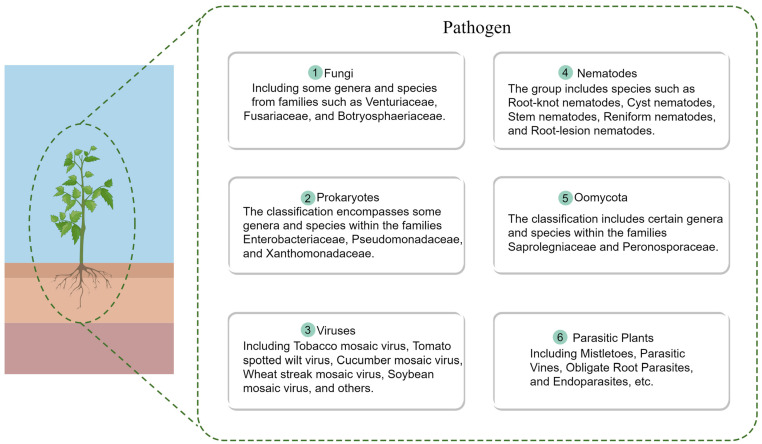
Categories of plant pathogens.

Plant pathogens encompass a vast array of categories, including Viruses, Prokaryotes, Fungi, Oomycota (such as *Albuginales* and *Peronosporaceae*), Nematodes, parasitic plants (like *Cuscuta* spp.) [18,19,20] (Figure 2).

It is known that there are more than 30,000 species of *Fungi* that cause plant diseases, accounting for approximately 70% to 80% of all plant diseases. They are distributed across multiple phyla, classes, orders, families, and genera, such as *Ascochyta*, *Didymella*, *Epicoccum*, and so on. *Oomycota* belong to the *Stramenopila* group; although morphologically similar to fungi, they are biologically distinct in terms of classification [21,22]. They are eukaryotic organisms but not classified as fungi and can cause diseases such as late blight caused by *Phytophthora* spp. [23]. Prokaryotes, including bacterial pathogens, can induce a variety of plant diseases, exemplified by *Pseudomonas syringae* [24].

Ahmed et al. [25] found that the interaction between mycorrhizal fungi and plants can affect plant–nematode interactions, thereby enhancing the plant’s resistance to nematodes, highlighting the potential of mycorrhizal fungi in improving plant resistance to diseases. Mycorrhizal fungi-induced disease resistance spans a variety of pathogen-induced diseases (Table 1). Mycorrhization by arbuscular mycorrhizal fungi (AMF) can significantly enhance the growth parameters, photosynthetic pigments, and flavonoid content in tomato plants, while reducing the severity and incidence of *Tomato mosaic virus* (ToMV) infection [26].

**Table 1:** Types of plant diseases inhibited by mycorrhizal fungi.

Pathogen	Disease Name	Pathogen	Host Plant	Mycorrhizal Fungal Names	Mycorrhizal Types	Mechanism of Disease Resistance
Oomycota	Late blight disease	*Phytophthora infestans*	*Solanum tuberosum*	*Glomus* sp.	Arbuscular mycorrhizae	Inducing systemic acquired resistance by activating plant defense genes (such as PR1 and PR2), reducing the leaf infection index, and enhancing resistance to late blight [23]
Fungi	Fusarium wilt	*Fusarium oxysporum*	*Solanum lycopersicum*	*Glomus mosseae*	Arbuscular mycorrhizae	Combating soil-borne pathogens in tomatoes, providing bioprotection effects [27]
		*Fusarium oxysporum*	*Salvia miltiorrhiza*	*Glomus versiforme*	Arbuscular mycorrhizae	Mycorrrhizal colonization enhances the host plant’s resistance to fungal pathogens by strengthening photosynthesis, root structure, and inducing the expression of defense enzymes and defense-related genes to combat infection [28]
		*Fusarium oxysporum*	*Musa acuminate*	*Rhizophagus irregularis*	Arbuscular mycorrhizae	Promoting the growth of banana plants, inducing the expression of defense-related genes, aiding in the suppression of wilt disease [29]
		*Fusarium oxysporum*	*Citrullus lanatus*	*Funneliformis mosseae* or *Glomus versiforme*	Arbuscular mycorrhizae	By inducing the root exudation of phthalic esters, altering soil enzyme activity and bacterial community composition, wilt disease in watermelon is mitigated [30]
	White Rot	*Erysiphe alphitoides*	*Quercus robur*	The commercially available mycorrhizal inoculant Ectovit^®^, which contains a variety of mycorrhizal fungi, was used	Multiple mycorrhizal fungi	Mycorrhizal fungi can significantly increase the levels of polyamines, soluble osmotic regulators (such as proline), and phenolic compounds in plant leaves, thereby enhancing the plant’s resistance to powdery mildew [31]
	Rust infection	*Melampsora larici-populina*	*Populus trichocarpa* × *deltoides*	*Hebeloma mesophaeum*	Ectomycorrhizae	By mitigating the reduction in the synthesis of phenolic compounds triggered by rust disease, the negative impact of rust on the host plant is compensated for [32]
	Verticillium wilt	*Rhizoctonia solani*	*Cucumis sativus*	*Glomus mosseae* and *Glomus clarum*	Arbuscular mycorrhizae	Mycorrhizal fungi significantly reduced disease severity and increased plant biomass, potentially through mechanisms such as improving nutritional status, reducing direct competition with pathogens, and inducing plant immunity [33]
		*Verticillium dahliae*	*Gossypium hirsutum*	*Glomus etunicatum*, *Glomus intraradices*, *Glomus versiforme*	Arbuscular mycorrhizae	Mycorrhizal fungi exhibit competitive interactions with the pathogen *V*. *dahliae*, which can alleviate the disease effects of *V*. *dahliae* on cotton and enhance the plant’s resistance to the disease [34]
		*Verticillium dahliae*	*Gossypium hirsutum*	*Rhizophagus irregularis*	Arbuscular mycorrhizae	By inducing the expression of plant resistance-related genes and the potential release of volatile compounds by mycorrhizal fungi symbionts, which directly affect the growth of pathogenic fungi [35]
	Early blight disease	*Alternaria solani*	*Solanum lycopersicum*	*Glomus intraradices*	Arbuscular mycorrhizae	Reducing the susceptibility of tomatoes to *A*. *solani*, diminishing disease symptoms, is akin to the induction of systemic resistance (ISR) [36]
	Damping off	*Rhizoctonia solani*	*Pinus tabulaeformis*	*Suillus laricinus*, *S*. *tomentosus*, *Amanita vaginata*, *Gomphidius viscidus*	Ectomycorrhizae	Inhibiting the growth of pathogens by producing hydrolytic enzymes (chitinase, β-1,3-glucanase, and β-glucosidase) that participate in the parasitic action on the fungi, altering the morphology of the pathogen [15]
	Black pod disease	*Phytophthora megakarya*	*Theobroma cacao*	*Gigaspora margarita* and *Acaulospora tuberculata*	Arbuscular mycorrhizae	Promoting the growth of cocoa, enhancing resistance to black pod disease, and increasing plant growth parameters, such as height, root, and stem weight, as well as phosphorus uptake [37]
	Black foot disease	*Cylindrocarpon macrodidymum*	*Vitis Rupestris*	*Glomus intraradices*	Arbuscular mycorrhizae	Reducing the susceptibility of grapevine roots to black foot disease, enhancing the plant’s resistance to abiotic or biotic stresses, and mitigating the severity of the disease [38]
	Decline syndrome	*Phytophthora cinnamomi*	*Quercus ilex*	*Tomentella* spp., *Russula* spp.	Ectomycorrhizae	Affecting the vitality of oak roots and the abundance of mycorrhizal fungi, the interplay of soil properties, topography, and root infection by *P*. *cinnamomi* influences the abundance of mycorrhizal fungi [7]
Prokaryotes	Bacterial wilt disease	*Ralstonia solanacearum*	*Solanum lycopersicum*	*Glomus mosseae*, *Scutellospora* sp., *Gigaspora margarita*	Arbuscular mycorrhizae	The integration of *Glomus mosseae* with the pathogen significantly enhanced the height and biomass of tomato plants, with no occurrence of disease symptoms [39]
		*Ralstonia solanacearum*	*Nicotiana tabacum*	*Glomus mosseae*	Arbuscular mycorrhizae	The combined application of *Trichoderma harzianum*-amended bio-organic fertilizer and the mycorrhizal fungus *Glomus mosseae* reduced the abundance of the pathogen, and increased the activities of polyphenol oxidase (PPO), phenylalanine ammonia-lyase (PAL), and peroxidase (POD) in the plants, promoting plant growth [40]
	Bacterial wilt	*Ralstonia solanacearum*	*Solanum lycopersicum*	*Glomus mosseae*	Arbuscular mycorrhizae	Tomatoes inoculated with *Glomus mosseae*, when combined with the use of organic fertilizers, have exhibited increased plant survival rates and yields [41]
		*Ralstonia solanacearum*	*Solanum lycopersicum*	*Rhizophagus irregularis*	Arbuscular mycorrhizae	By activating the plant’s defense mechanisms [42]
Nematodes	Root-knot nematode disease	*Meloidogyne incognita*	*Solanum lycopersicum*	*Glomus mosseae*	Arbuscular mycorrhizae	Inducing systemic acquired resistance reduced the number of root-knot nematodes, mitigating their damage to tomato root systems [43]
	Root-lesion nematode disease	*Pratylenchus penetrans*	*Solanum lycopersicum*	*Glomus mosseae*	Arbuscular mycorrhizae	Inducing systemic resistance significantly reduced the number of root-lesion nematodes, decreased their reproduction rate, and lessened the damage to tomato root systems [43]
	False root-knot nematode	*Nacobbus aberrans*	*Solanum lycopersicum*	*Glomus intraradices*	Arbuscular mycorrhizae	Reducing root damage caused by nematodes (decreasing the number of root galls) and inhibiting nematode reproduction [44]
Viruses	Tomato yellow leaf curl disease	Tomato yellow leaf curl Sardinia virus (TYLCSV)	*Solanum tuberosum*	*Funneliformis mosseae*	Arbuscular mycorrhizae	Mitigating the severity of viral symptoms, reducing the concentration of viral DNA in tomatoes, and enhancing the tolerance of tomatoes to TYLCSV [45]

Simultaneously, in the context of fungal diseases, mycorrhizal fungi have been observed to enhance the resistance of plants to *Fusarium* wilt diseases caused by specific pathogenic *Fusarium* species. These pathogenic species, when infecting plants, can typically result in symptoms such as root rot and desiccation of the plant body [46]. Moreover, mycorrhizal fungi can also strengthen the plant’s defense against root rot caused by *Rhizoctonia* spp., a disease that is widespread in many crops and causes necrosis of the roots and stunted growth of the plant [47]. Regarding bacterial diseases, mycorrhizal fungi have also been demonstrated to reduce the incidence of leaf spot and canker diseases caused by bacteria such as *Pseudomonas syringae* [48].

### 2.2. The Molecular Mechanisms Underlying the Activation of Plant Disease Resistance by Mycorrhizal Fungi

The molecular mechanisms by which mycorrhizal fungi induce plant disease resistance involve a multitude of molecular changes and signal transduction pathways. Upon contact with plant roots, mycorrhizal fungi secrete signaling molecules [49], such as short-chain fatty acids and sugars, which are recognized by receptors on the root surface, initiating internal signal transduction pathways within the plant [50,51]. During this process, changes in intracellular calcium ion concentrations activate calcium-binding protein kinases (CDPKs), which further phosphorylate a series of downstream signaling molecules [52].These signaling molecules may include jasmonate synthases (JAS), which, under the action of CDPKs, convert precursor substances into jasmonic acid. As a signaling molecule, jasmonic acid activates its downstream signaling pathways, including jasmonic acid receptors (e.g., COI1) and related signaling molecules. The activation of the jasmonic acid signaling pathway leads to the phosphorylation and activation of specific transcription factors (e.g., MYC2). The activated transcription factors enter the nucleus and regulate the expression of genes related to disease resistance, including pathogenesis-related proteins (PR proteins) and other defense-related genes [53,54,55,56,57]. The expression of these genes enhances the plant’s defense mechanisms, including the reinforcement of cell walls, the production and activity of pathogen-associated proteins, and the generation of reactive oxygen species.

The presence of mycorrhizal fungi activates the antioxidant system within plants, potentially enhancing the activity of antioxidant enzymes such as cytosolic ascorbate peroxidase (cAPX), superoxide dismutase (SOD), peroxidase (POD), and glutathione reductase (GR) [58]. These enzymes help to eliminate reactive oxygen species produced during pathogen invasion, with cAPX being a primary scavenger of reactive oxygen. cAPX catalyzes the dehydrogenation reaction of H_2_O_2_ with ascorbate residues, converting H_2_O_2_ into water, thereby protecting plant cells from oxidative damage [59]. Phosphatases further enhance the plant’s antioxidant capacity by regulating the phosphorylation state of these antioxidant enzymes [60]. Hashem et al. [61] demonstrated that AMF can enhance plant disease resistance by promoting the activity of phosphatases. In this study, inoculation with AMF not only increased the activity of plant phosphorus metabolism-related enzymes but also enhanced the defense system by increasing chlorophyll content and improving water status, combating oxidative stress caused by *Fusarium oxysporum* f. sp. lycopersici (FOL). Furthermore, AMF can elevate the activity of the antioxidant enzyme system, which helps to eliminate reactive oxygen species and protect plants from oxidative damage, ensuring an effective response to pathogen attacks.

Mycorrhizal fungi also increase the absorption area of plant roots for nutrients in the soil through their mycelial network [62], especially key nutrients such as phosphorus and nitrogen, which are essential for the plant immune system [63]. Phosphatases can catalyze the hydrolysis of organic phosphorus compounds in the soil, releasing inorganic phosphate that is readily available for plant uptake, thereby enhancing the nutritional status of the plant [64]. This process not only improves the nutritional status of the plant but may also regulate the levels of plant hormones [65], such as salicylic acid (SA) and jasmonic acid (JA), indirectly enhancing the plant’s disease resistance.

In summary, the molecular mechanisms by which mycorrhizal fungi induce plant disease resistance involve a series of molecular changes at various levels, from signal recognition and transduction to gene expression regulation and the activation of the antioxidant system. Phosphatases play a key regulatory role in this process, enhancing plant resistance by finely tuning signal transduction pathways and gene expression.

## 3. The Role of Phosphatases in the Induction of Plant Disease Resistance by Mycorrhizal Fungi

Phosphatases play a multifaceted regulatory role in the plant disease resistance induced by mycorrhizal fungi, not only participating in the activation and modulation of signaling pathways, but also affecting the levels of plant hormones and the activity of the antioxidant system, together forming a complex defense network for plants against pathogens.

### 3.1. The Decomposition of Insoluble and Sparingly Soluble Phosphorus in the Soil

In soil, inorganic phosphorus, particularly that in mineral form, is readily adsorbed and fixed by soil particles, resulting in plant-available free phosphate concentrations typically below 10 µmol/L [66]. In acidic soils, phosphorus predominantly exists as iron-bound, aluminum-bound, and occluded forms, while in calcareous soils, it is mainly present in the form of insoluble calcium phosphates.

Mycorrhizal fungi, through their extensive mycelial networks, can expand the contact area between plant roots and soil [67], enhancing the activation and absorption of soil phosphorus [68,69]. Under acidification conditions, AMF play a crucial role in soil phosphorus cycling and plant nutrition, participating in the regulation of soil pH and the availability of phosphorus through various mechanisms. For instance, AMF can secrete organic acids and other chelating agents that compete with iron and aluminum ions in the soil, reducing the adsorption and fixation of phosphorus, thereby releasing more available phosphorus for plant uptake [70]. Simultaneously, mycorrhizal fungi secrete phosphatases that catalyze the hydrolysis of organic phosphorus compounds in the soil, thereby facilitating the dissolution and mineralization of insoluble phosphorus [71,72,73,74,75,76,77,78]. Consequently, phosphatases are considered key factors affecting the nutritional exchange between mycorrhizal fungi and their plant hosts [79].

Improved phosphorus nutrition promotes the growth of plant root systems, aiding in the adaptation to environmental stresses, such as drought and saline–alkali conditions, and enhances resistance to pathogens [80,81].

### 3.2. Involvement in the Regulation of Plant Defense and Immune Responses

Pattern recognition receptors (PRRs) on the surface of plant root cells perceive pathogen-associated molecular patterns (MAMPs), triggering an initial immune response known as pattern-triggered immunity (PTI) [82,83].

**Figure 3:**
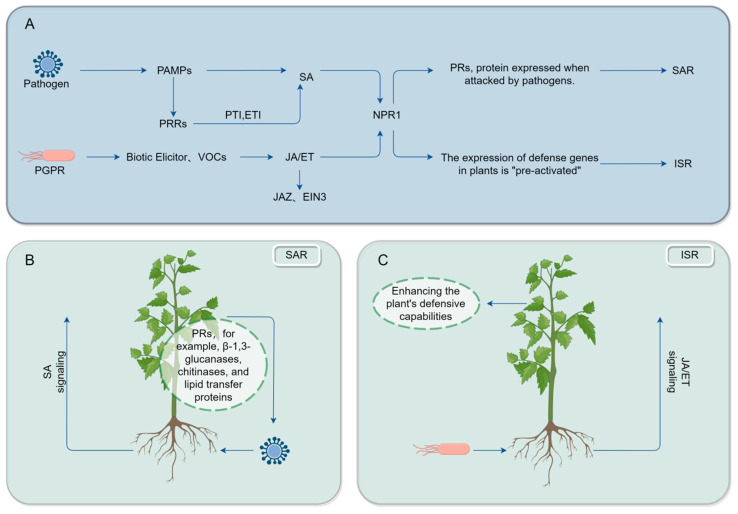
Plant root and shoot defense initiation mechanism. (**A**) The mechanisms underlying induced systemic resistance (ISR) and systemic acquired resistance (SAR) involve distinct yet interconnected pathways. (**B**) Systemic acquired resistance represents an immune state established throughout the plant following local infection. SAR is primarily activated through the salicylic acid (SA) signaling pathway, endowing the plant with enhanced resistance to subsequent pathogen infections. (**C**) Induced systemic resistance is an immune response triggered by beneficial microbes or chemical substances perceived by the plant’s roots. This response is activated through signaling pathways mediated by plant hormones such as jasmonic acid (JA) and ethylene (ET), strengthening the plant’s defense against a broad spectrum of pathogens. Abbreviations: EIN3, Ethylene Insensitive 3; JAZ, Jasmonate Zim-Domain Protein; PAMPs, pathogen-associated molecular patterns; PRRs, pattern recognition receptors; JA, jasmonic acid; ET, ethylene; PRs, pathogenesis-related proteins; SA, salicylic acid; NPR1, non-race-specific disease resistance 1.

PTI involves the activation of intracellular signaling pathways, including the opening of calcium ion channels, the activation of protein kinases, and the production of reactive oxygen species (ROS), leading to the upregulation of defense-related gene expression, such as PR proteins and antimicrobial peptides [84,85,86], as well as the significant enhancement of defense-related genes (PR2a, PAL, and AOS) and the key gene BX9 in the DIMBOA (2,4-Dihydroxy-7-methoxy-2H-1,4-benzoxazin-3(4H)-one) biosynthetic pathway [87]. During the process of mycorrhiza formation, plant defense responses and molecular reprogramming are modulated to effectively activate the plant’s immune response and the expression of defense genes, which is highly similar to induced systemic resistance (ISR) [88,89] (Figure 3). This induced resistance is termed mycorrhiza-induced resistance (MIR). Phosphatases play a crucial role in ISR [90,91] and programmed cell death (PCD) by regulating the phosphorylation status of related proteins [92].

Phosphatases are primarily derived from fungal hyphae and hyphal sheaths (fungal cellular structures enveloping plant root tips), functioning by removing phosphate groups from signaling molecules [93]. It can modulate the activity and stability of immune-related proteins in plants, control the intensity and duration of signaling, and prevent excessive immune responses, thereby influencing plant’s pattern-triggered immunity (PTI) and effector-triggered immunity (ETI) responses [83].

**Figure 4:**
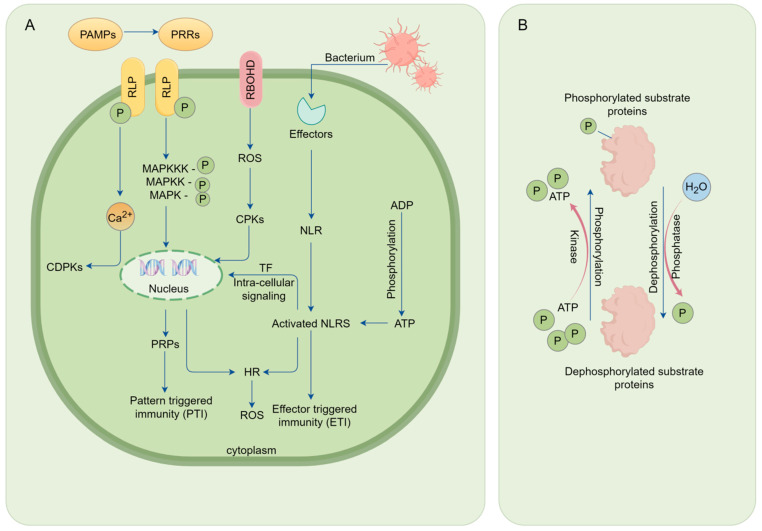
The immune response process of plant root systems against pathogenic agents. (**A**) Pattern-triggered immunity (PTI) process: Plant cells recognize pathogen-associated molecular patterns (PAMPs) through pattern recognition receptors (PRRs), which initiate a series of downstream signaling pathways. This includes the activation of mitogen-activated protein kinase kinase kinases (MAPKKKs), which in turn activate mitogen-activated protein kinase kinases (MAPKKs) and mitogen-activated protein kinases (MAPKs). The activation of these kinases leads to the conversion of ADP to ATP, providing energy for signal transduction. The signaling pathways activate calcium-dependent protein kinases (CDPKs) and other protein kinases, which further phosphorylate substrate proteins, facilitating intracellular signal transduction. These signals ultimately lead to the activation of transcription factors (TFs) in the nucleus, thereby inducing the expression of pathogenesis-related proteins (PRPs) and enhancing the plant’s defense response. Effector-triggered immunity (ETI) process: To combat PTI, pathogens secrete effector proteins. The recognition of these effectors by NLR proteins leads to their activation, which may involve oligomerization or conformational changes. The activation of NLR proteins initiates a potent immune response, characterized by intracellular signal transduction and changes in gene expression. This results in the binding of the activated NLR proteins to the effectors, inducing programmed cell death, such as the hypersensitive response (HR). (**B**) In the process of intracellular signal transduction in plant cells, the phosphorylation and dephosphorylation of proteins occur. This process is crucial for the regulation of various cellular functions and responses to environmental stimuli. Abbreviations: ROS, reactive oxygen species; CDPKs, calcium-dependent protein kinases; PPRs, pentatricopeptide repeats; TF, transcription factor; NB, nucleotide-binding site; LRR, leucine-rich repeat; PRPs, pathogenesis-related proteins; NLR, nucleotide-binding leucine-rich repeat; CPKs, calcium-dependent protein kinases; RBOHD, Respiratory Burst Oxidase Homologue D; RLP, receptor-like protein; NLRs, nucleotide-binding leucine-rich repeats.

In the intricate network of plant immunity, the mitogen-activated protein kinase (MAPK) cascade is a central signaling pathway. The activity of the MAPK can promote a local cell death mechanism known as the hypersensitive response (HR), which is part of the plant’s defense strategy [94]. Phosphatases regulate the PTI and ETI responses by dephosphorylating key components of the MAPK cascade, such as MAPKKK, MAPKK, and MAPK (Figure 4). This regulation not only affects the transmission of immune signals but also involves the activity and stability of specific transcription factors, such as WRKY transcription factors, thereby influencing the expression of genes related to SAR [95,96]. After the initial infection, WRKY transcription factors activate downstream resistance genes, helping the plant to establish systemic resistance in tissues distant from the site of primary infection [97]. Among them, the transcription factor WRKY33 is activated by the MPK3/6 kinase and directly binds to the promoter of the ALD1 gene, regulating its expression. The enzyme encoded by the ALD1 gene is responsible for the synthesis of pipecolic acid, a key mobile signal molecule in SAR [98].

Wei et al. [99] found that the phosphatase C-terminal domain phosphatase-like 1 (CPL1) plays a negative regulatory role in plant immunity, enhancing the plant’s immune response. CPL1 is localized in the nucleus and can interact with MKK4, MKK5, MPK3, and MPK6, disrupting the interaction between MKK4/MKK5 and MPK3/MPK6, and weakening the transmission of immune signals, thus negatively regulating *Arabidopsis* resistance to bacteria. This interference depends on the phosphatase activity of CPL1, revealing a new function of phosphatases in the regulatory network of plant immunity.

In addition, 2C-type protein phosphatases (PP2Cs) have been identified as key regulators of the antiviral defense mechanism [100,101,102]. Diao et al. [103] found that PP2C15 acts as a negative regulator of plant immunity by targeting the BRI1-associated receptor kinase 1 (BAK1). Among the 56 PP2Cs, 14 significantly suppressed the immune response induced by flg22, a bacterial pattern recognition molecule, with PP2C15 negatively regulating the immune response by interacting with BAK1 and dephosphorylating it.

Hence, it is clear that phosphatases play a critical role in modulating various aspects of plant defense and immune responses, such as signal transduction, regulation of transcription factor activity, and control of PR gene expression, making them an indispensable component of the plant’s disease resistance regulatory network.

### 3.3. The Interplay with Hormonal Signaling

Plant hormones such as jasmonic acid (JA), salicylic acid (SA), auxin, and gibberellin (GA) are pivotal signaling molecules in the plant immune system. The transcription factor OsARF17, a key player in the auxin signaling pathway, is implicated in the regulation of plant responses to various biotic stresses, including viral infections [104]. Plants may balance immune responses and growth through such hormonal crosstalk. Specifically, the salicylic acid receptor NPR1, as a subunit of the ubiquitin E3 ligase, can promote the polyubiquitination and degradation of the GA receptor GID1, enhancing the stability of DELLA proteins, which are negative regulators of the GA signaling pathway [105]. Meanwhile, CPL3, a phosphatase associated with the GA response, interacts with DELLA proteins and is involved in the phosphorylation of RNA polymerase II, playing an important role in the regulation of plant growth and development [106]. Phosphatases may also enhance the systemic resistance of plants by regulating the SA and JA signaling pathways [107,108]. For example, by participating in the synthesis, signal transduction, or response of proteins involved in SA, they can affect plant resistance to diseases. This regulatory effect may involve the phosphorylation status of multiple levels of proteins in the SA signaling pathway [109], thereby affecting the plant’s recognition of pathogens, signal amplification, and the activation of the final defense response [103]. In addition to SA, other oxylipins may also be involved in signal transduction during the interaction between mycorrhizal fungi and pathogens in the roots [110].

It can be seen that the roles of plant hormones and phosphatases in the plant immune system are interconnected and interdependent. Together, they form a complex regulatory network that interacts with other hormonal signaling pathways, either directly or indirectly, and participates in the plant’s defense mechanisms against pathogens.

### 3.4. Restriction the Invasion and Spread of Pathogens

Fernandes et al. [111] have discovered that *Fusarium oxysporum* can induce rapid alkalinization of the extracellular space in the host by secreting a functional homologue of the plant’s Rapid ALkalinizing Factor (RALF), known as the F-RALF peptide. This alkalization effect is crucial for the pathogen’s infection process, as it not only facilitates the invasive growth of the fungus but also activates cellular signaling pathways associated with pathogenicity, thereby enhancing its virulence.

Specifically, such alkalinization can shift the pH milieu within host tissues, thereby providing a more propitious environment for pathogen proliferation. Additionally, this alkalization may indirectly impact the functionality of the host cells, including intracellular signaling pathways, potentially aiding the efficacy of effector proteins secreted by the pathogen to manipulate the host’s cellular machinery. However, phosphatases secreted by mycorrhizal fungi can dephosphorylate these effector proteins, reducing their activity and thereby limiting the infection and spread of pathogens [112,113,114]. In addition, specific phosphatases secreted by mycorrhizal fungi can hydrolyze polysaccharides in the pathogen cell wall, weakening the pathogen’s ability to infect [115], and activating the plant’s immune response, providing an effective defense mechanism for the plant [116].

Simultaneously, there is competition between mycorrhizal fungi and pathogens for resources around the plant roots, which helps to restrict the growth and spread of pathogens. Mycorrhizal fungi can reduce the chances of pathogen infection through competition and antagonism. Phosphatases may also interfere with the quorum-sensing mechanism of pathogens by hydrolyzing quorum sensing signal molecules, such as AHLs (N-acyl homoserine lactones) [117], reducing the production of virulence factors by pathogens and thus decreasing their pathogenicity to the host.

### 3.5. The Interaction between Mycorrhizal Fungi and Beneficial Microorganisms

#### 3.5.1. Mycorrhizal Fungi and Beneficial Microbes Synergize to Enhance Crop Disease Resistance

Caravaca et al. [118] found that the *Streptomyces* AcH505 strain enhances the growth of oak trees by increasing the abundance of saprotrophic and ectomycorrhizal fungi in the rhizosphere (an increase of 158% compared to the control group), which can counteract the damage caused by nematodes by promoting the growth of oak micro-cuttings. This indicates that mycorrhizal fungi form a symbiotic relationship with plant roots, which not only promotes the absorption of nutrients by the plant but also changes the composition of the microbial community in the plant’s rhizosphere [119,120,121,122,123,124]. Through interactions with various beneficial microorganisms, they inhibit the activity of pathogens and jointly enhance the plant’s disease resistance [125,126,127,128] (Table 2).

Beneficial microorganisms can promote the formation of mycorrhizae by secreting compounds that stimulate the growth of mycorrhizal fungi, such as hormones, enzymes, and organic acids [129,130,131]. At the same time, they can also degrade toxic substances in the soil [132], protecting mycorrhizal fungi from the effects of harmful substances. During the interaction process, mycorrhizal fungi and these beneficial microorganisms can inhibit the growth of pathogens by producing antibiotics, siderophores, and SAR signaling molecules, improve the nutritional status of plants, activate various defense-related genes in plants, and change the composition of root exudates to regulate the plant’s sensitivity to pathogens, thereby enhancing the plant’s physiological health and resistance to environmental stress, and thus improving the plant’s resistance to pathogens [133,134,135,136,137,138,139].

**Table 2:** Mechanisms by which mycorrhizal fungi synergize with beneficial microbes to enhance crop resistance to diseases.

Disease Name	Pathogenic Microorganism	Beneficial Microbial Species	Host Plant	Mycorrhizal Fungal Names	Mycorrhizal Types	Synergistic Mechanism
Bacterial wilt	*Ralstonia solanacearum*	*Trichoderma harzianum*	*Nicotiana tabacum*	*Glomus mosseae*	Arbuscular mycorrhizae	The synergistic action of these two factors has led to a reduction in the abundance of soil-borne pathogenic microorganisms and a concomitant enhancement of the activity of plant systemic resistance-related enzymes [40]
	*Ralstonia solanacearum*	*Bacillus* spp., *Pseudomonas* spp., *Azotobacter* spp.	*Solanum tuberosum*	*Glomus intraradices*, *G*. *etunicatum*, *G*. *mosseae*	Arbuscular mycorrhizae	The combination of biocontrol agents (BCA) and arbuscular mycorrhizal fungi (AMF) may mitigate disease severity through antagonistic interactions and influence the microbial community by altering root exudates [140]
Spring black stem and leaf spot	*Phoma medicaginis*	*Sinorhizobium medicae*	*Medicago sativa*	*Funneliformis mosseae*	Arbuscular mycorrhizae	The mutual promotion between mycorrhizal fungi and other microbes enhances the formation of root nodules and mycorrhizal colonization, which in turn boosts the plant’s phosphorus and nitrogen uptake. This interaction also augments the activity of plant defense compounds and enzymes, consequently reducing the disease index [141]
Anthracnose	*Colletotrichum orbiculare*	*Phoma* sp., *Penicillium simplicissimum*	*Cucumis sativus*	*Glomus mosseae*	Arbuscular mycorrhizae	The interplay between Plant growth-promoting fungi (PGPF) and arbuscular mycorrhizal fungi (AMF) may influence the level of disease protection through competitive interactions for space or nutrients [142]
Fusarium wilt	*Fusarium oxysporum*	*Trichoderma harzianum*	*Cucumis melo*	*Glomus constrictum*, *G*. *mosseae*, *G*. *claroideum*, *G*. *intraradices*	Arbuscular mycorrhizae	*Trichoderma harzianum* and arbuscular mycorrhizal fungi (AMF) may synergistically control diseases by enhancing nutrient uptake and inducing systemic resistance in plants [143]
Bipolaris sorokiniana	*Bipolaris sorokiniana*	*Epichloë festucae*	*Lolium perenne*	*Claroideoglomus etunicatum*	Arbuscular mycorrhizae	Endophytic fungi and mycorrhizal fungi enhance the resistance of plants to diseases by activating defense-related enzymes, increasing the activity of plant hormones, and elevating the content of lignin [144]
Root rot and charcoal rot	*Macrophomina phaseolina*	*Brettanomyces naardensis*	*Helianthus annuus*	*Acaulospora bireticulata*	Arbuscular mycorrhizae	Yeasts facilitate the development of arbuscular mycorrhizal fungi (AMF) by supplying vitamin B12. The combined action of these two organisms alters the pattern of root exudates, impacting the plant’s rhizosphere microbial community and inhibiting the invasion and growth of pathogenic fungi [145]
Late blight	*Phytophthora infestans*	*Pseudomonas* sp.	*Solanum tuberosum*	*Rhizophagus irregularis*	Arbuscular mycorrhizae	The co-inoculation of plant growth-promoting microbes and arbuscular mycorrhizal fungi may activate the plant’s systemic defense system, leading to the upregulation of ethylene response factor 3 (ERF3) and thereby enhancing the plant’s resistance to diseases [146]
Take-all	*Gaeumannomyces graminis*	*Pseudomonas fluorescens*	*Triticum aestivum*	*Glomus mossea*	Arbuscular mycorrhizae	Mycorrhizal fungi enhance the plant’s resistance to diseases, while beneficial microbes influence plant metabolism or directly inhibit pathogen growth through their metabolic byproducts [147]
Root-knot nematode disease	*Meloidogyne incognita*	*Bacillus polymyxa*, *Bacillus* sp.	*Solanum lycopersicum*	*Glomus versiforme*, *Glomus mosseae*	Arbuscular mycorrhizae	Beneficial microbes augment the colonization of arbuscular mycorrhizal (AM) fungi in the roots, and in turn, AM fungi enhance the population of beneficial microbes in the rhizosphere; together, they suppress nematode damage and promote plant growth [148]
Sphaeropsis Shoot Blight	*Sphaeropsis sapinea*	*Bacillus pumilus*	*Pinus thunbergii*	*Hymenochaete* sp. Rl	Ectomycorrhizae	Mycorrhizal fungi elicit systemic defense responses in plants. In concert, beneficial bacteria facilitate the formation of symbiotic structures between the mycorrhizal fungi and their host plants [149]
White rot	*Sclerotinia sclerotiorum*	PGPR	*Fragaria*	AMF	Arbuscular mycorrhizae	The combined application of mycorrhizal fungi and plant growth-promoting rhizobacteria (PGPR) enhances plant biomass, promotes vegetative growth, and reduces disease indices [150]

#### 3.5.2. The Role of Phosphatases in the Synergistic Process

Abdel-Fattah et al. [151] found that *Sorghum bicolor* inoculated with the arbuscular mycorrhizal fungus *Glomus intraradices* no. LAP8 had significantly higher acid and alkaline phosphatase activities in root extracts compared to non-mycorrhizal plants not inoculated with the fungus. The increase in phosphatase activity leads to the enhanced availability of phosphorus in the soil [152], which can improve soil fertility [153], create more suitable living conditions for beneficial microbes, provide more nutrients, promote the growth and metabolic activities of beneficial microbes, and thus affect the structure and diversity of the soil microbial community [154,155].

Furthermore, the enhancement of phosphatase activity can protect the host plant from changes induced by ionic and osmotic stress [156], and promote the growth and extension of mycorrhizal fungal hyphae [157], thereby increasing the opportunities for contact with other beneficial microbes in the soil, forming a more complex microbial network, and enhancing the plant’s disease resistance.

Thus, the activity of phosphatases is closely related to soil fertility, the symbiotic relationship with mycorrhizal fungi, and the structure and function of the soil microbial community.

## 4. Perspectives

Current research collectively underscores the pivotal role of mycorrhizal fungi in the absorption and translocation of essential nutrients within symbiotic relationships. Mycorrhizal fungi enhance plant immunity by improving the nutritional status of the host, particularly in terms of phosphorus uptake. Moreover, the mycelial networks formed by mycorrhizal fungi facilitate the exchange of materials and signal transmission among plants, aiding in the collective defense against diseases within plant communities.

The resistance conferred by mycorrhizal fungi through the action of phosphatases is a relatively complex process. Although phosphatases themselves do not directly combat pathogenic organisms, they contribute to the plant defense system by modulating plant hormone signaling pathways, enhancing nutritional status, disrupting the quorum-sensing mechanisms of pathogens, and activating plant immune responses. The role of phosphatases in immune regulation is multifaceted; they are involved not only in the fundamental physiological processes of the plant but also in the response and modulation to pathogen attacks. However, the mechanisms underlying resistance remain contentious, necessitating further integration of molecular techniques and physiological experiments to elucidate the specific mechanisms by which phosphatases enhance plant disease resistance in mycorrhizal associations.

The involvement of phosphatases in induced plant resistance encompasses a variety of signaling molecules and metabolic pathways, providing significant clues for further dissection of the molecular mechanisms underlying plant-microbe interactions. However, the specific roles and molecular mechanisms of phosphatases in different mycorrhizal fungi and plant systems remain to be further elucidated. Additionally, how phosphatases interact with other plant defense signaling pathways and how these interactions influence plant resistance to various pathogens are key points for future research. Furthermore, the impact of environmental factors such as soil pH, nutrient status, and climate change on the secretion of phosphatases by mycorrhizal fungi, and how these factors regulate plant resistance, are also important directions for future investigation.

Further research in this field can pave new avenues and methods for plant disease control and the study of mycorrhizal fungi, offering valuable insights for sustainable strategies to enhance crop productivity.

## Data Availability

All data analyzed during this study are included in this article.
